# Investigating the role of relationship satisfaction and paternal psychological distress during pregnancy on offspring health in early life

**DOI:** 10.1192/bjo.2023.59

**Published:** 2023-05-25

**Authors:** Laura Korhonen, Saara Nolvi, Ville Peltola, Minna Lukkarinen, Riikka Korja, Linnea Karlsson, Hasse Karlsson

**Affiliations:** FinnBrain Birth Cohort Study, Turku Brain and Mind Center, Department of Clinical Medicine, University of Turku, Finland; and Department of Paediatrics and Adolescent Medicine, Turku University Hospital and University of Turku, Finland; FinnBrain Birth Cohort Study, Turku Brain and Mind Center, Department of Clinical Medicine, University of Turku, Finland; and Turku Institute for Advanced Studies, Department of Psychology and Speech-Language Pathology, University of Turku, Finland; Department of Paediatrics and Adolescent Medicine, Turku University Hospital and University of Turku, Finland; FinnBrain Birth Cohort Study, Turku Brain and Mind Center, Department of Clinical Medicine, University of Turku, Finland; FinnBrain Birth Cohort Study, Turku Brain and Mind Center, Department of Clinical Medicine, University of Turku, Finland; Department of Paediatrics and Adolescent Medicine, Turku University Hospital and University of Turku, Finland; and Centre for Population Health Research, University of Turku and Turku University Hospital, Finland; FinnBrain Birth Cohort Study, Turku Brain and Mind Center, Department of Clinical Medicine, University of Turku, Finland; Centre for Population Health Research, University of Turku and Turku University Hospital, Finland; and Department of Psychiatry, Turku University Hospital and University of Turku, Finland

**Keywords:** Psychological distress, parental relationship satisfaction, child health, perinatal psychiatry, neuroendocrinology

## Abstract

**Background:**

The research on the role of father in the foetal programming of health and behaviour has received increasing attention. However, the influences of paternal depressive symptoms and couple relationship satisfaction during pregnancy – potentially mediated via maternal well-being – on the offspring's risk of infections in early life is still seldom assessed.

**Aims:**

The aim was to investigate if paternal psychological distress during pregnancy is associated with elevated risk of recurrent respiratory infections (RRIs) for offspring at 12 months of age, and whether maternal distress mediates the association between paternal distress and offspring RRIs.

**Method:**

The study population was drawn from the nested case–control cohort of the FinnBrain Birth Cohort Study. Children with RRIs (*n* = 50) were identified by maternal reports at the age of 12 months, whereas mothers did not report RRIs for the comparison group (*n* = 716). Parental depressive symptoms were measured with the Edinburgh Postnatal Depression Scale and couple relationship satisfaction was measured with the Revised Dyadic Adjustment Scale.

**Results:**

The association between paternal depressive symptoms during pregnancy and offspring RRIs was mediated by maternal prenatal depressive symptoms. Additionally, paternal poorer relationship satisfaction was associated with child RRIs independently of maternal distress.

**Conclusions:**

The results suggest different pathways through which paternal distress during pregnancy may contribute to elevated risk of offspring RRIs, and more research is needed to study their underlying mechanisms. Paternal distress and couple relationship satisfaction during pregnancy should be assessed and screened as a contributor to offspring health.

## Background

Recurrent respiratory infections (RRIs) in children have a significant impact on society. RRIs have associated costs, as children need several antibiotic treatments and may undergo small surgical procedures such as insertion of tympanostomy tubes.^1,2^ Because of these infections, parents are absent from work and have lower quality of life.^3^ Known risk factors for childhood RRIs are parental smoking, prematurity, male gender, lower socioeconomic status of the family, shorter duration of breastfeeding, greater number of siblings and attendance at daycare.^4–6^ In addition, prenatal maternal psychological distress has been established as a risk factor for offspring RRIs.^7,8^ Early-life stress, comprising diverse sources of both prenatal and postnatal stressors including parental distress, potentially has a programming effect – that is, the ability to impose changes in individual physiology, metabolism and epigenetics and subsequent health and disease risk.^7,9^

During pregnancy, the parental relationship is typically one of the main sources of psychological support for both adults. Therefore, the programming effects of maternal prenatal distress may be explained not only by mothers’ individual well-being, but also through distress experienced within the family. Still, few studies in the field have taken into account the role of paternal distress on offspring health, including RRIs.^10^ The possible influence of paternal distress on offspring health and development may be transmitted through maternal well-being during pregnancy.^11^ In previous literature, maternal well-being has been identified as a fundamental factor for child health and development.^12–16^ Recently, some studies have reported associations between both parents’ psychosocial distress and child poorer physical health outcomes, including wheezing and RRIs,^15,16^ whereas others have reported no direct association between paternal distress during pregnancy and child physical health outcomes.^16–18^ This could point to a complex and multifactorial pattern of paternal influences on child health during pregnancy. One possibility stemming from research focused on child socioemotional development is that the influence of paternal psychosocial distress during pregnancy on child health outcomes may be transmitted through maternal well-being, and more specifically, through couple relationship satisfaction and conflict,^19,20^ which may then interfere with adaptation to parenting postnatally, and thus child health outcomes.^21^ However, to our knowledge, there are no studies that have examined the role of paternal distress and its transmission through maternal prenatal well-being in terms of child physical health outcomes, such as RRIs.

## Study aims

We investigated whether the depressive symptoms and relationship satisfaction experienced by the father during pregnancy are associated with offspring RRIs by 12 months of age (measured as a binary variable with either recurrent infections or no recurrent infections). Primarily, we hypothesised that fathers’ depressive symptoms and couple relationship satisfaction during pregnancy would be linked with offspring RRIs through maternal depressive symptoms and/or relationship satisfaction during pregnancy. First, to establish the dependency of paternal symptoms on maternal well-being, we tested whether paternal symptoms were associated with child RRIs independently of maternal prenatal and postnatal distress symptoms. Next, we examined whether maternal depressive symptoms and/or relationship satisfaction mediated the association between paternal depressive symptoms and relationship satisfaction, and the risk of offspring RRIs. The study is based on a previous study in the same cohort that reported an association between maternal distress and relationship satisfaction during pregnancy and offspring RRIs.^8^ Here, we extend this by examining the role of paternal distress and its transmission to offspring risk for RRIs in a subsample of this previous study.

## Method

### Participants

The FinnBrain Birth Cohort Study (www.finnbrain.fi) is a birth study investigating the effects of prenatal and early-life stress exposure on child health.^22^ The families were recruited by research nurses when attending the free-of-charge ultrasounds at 12 gestational weeks in three maternity welfare clinics in the Southwestern Hospital District and Åland Islands in Finland during December 2011 to April 2015. The baseline cohort consisted of 3808 women, their 3837 babies and 2623 fathers/partners. Mothers and fathers were considered eligible to participate in the study if they had a verified pregnancy and sufficient knowledge of Finnish or Swedish (the official languages of Finland) to fill in the study questionnaires.

**Fig. 1:**
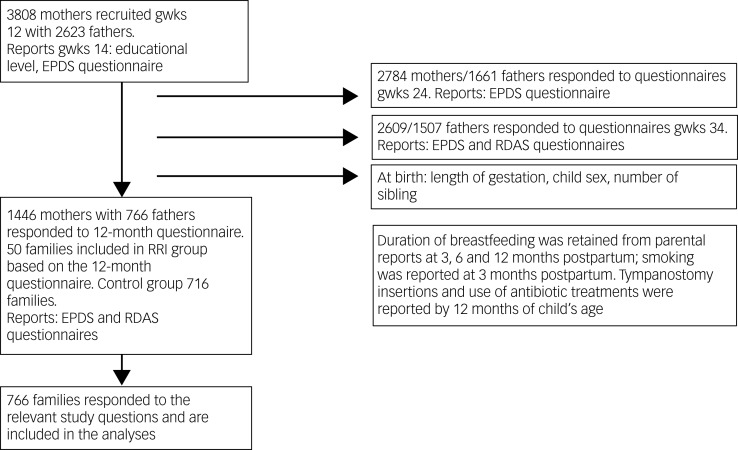
Timeline of data collection. gwks, gestational weeks; EPDS, Edinburgh Postnatal Depression; RDAS, Revised Dyadic Adjustment Scale; RRI, recurrent respiratory infections.

The present study comprised families in which both the mother and father had reported depressive symptoms at least twice during pregnancy, as well as child RRIs at 12 months of age. No additional criteria for inclusion or exclusion were used. This resulted in *N* = 766 families (Fig. 1). Compared with the fathers who did not respond to all questionnaires, the fathers with complete data were older (*t*(2605) = −4.64, *P* < 0.001), had better economic satisfaction (*t*(1584) = −2.58, *P* = 0.01) and had a higher educational level (*t χ*^2^(2) = 33.92, *P* < 0.011). However, there were no significant differences between the responders and non-responders regarding depressive symptoms or relationship satisfaction during pregnancy (*P* > 0.09).

Participating parents gave written informed consent for themselves and on behalf of the expected child. The authors assert that all procedures contributing to this work comply with the ethical standards of the relevant national and institutional committees on human experimentation and with the Helsinki Declaration of 1975, as revised in 2008. All procedures involving human patients were approved by the Ethics Committee of the Hospital District of Southwest Finland (identifier: ETMK:57/180/2011).

### Measures

See Fig. 1 for the timeline of data collection and attrition at each time point. The research questionnaires were either mailed to the participants or filled out online according to each participating parent's choice. The parents reported their depressive symptoms at 14, 24 and 34 gestational weeks, and relationship satisfaction at 34 gestational weeks. Additionally, both parents reported depressive symptoms and couple relationship satisfaction at 12 months postpartum. Finally, parents reported child RRIs at 12 months of age.

Children with RRIs were identified from maternal reports on the question ‘Has your child had recurrent infections?’ (yes/no) at the age of 12 months.^8^ In the study sample, RRIs by 12 months of age were reported in 50 out of 766 infants (6.5%); 716 out of 766 (93.5%) infants had no RRIs during the follow-up, and formed the comparison group. The dichotomic variable was used as the main response variable in the analysis.

#### Paternal and maternal depressive symptoms

Parental depressive symptoms were assessed with the Edinburgh Postnatal Depression Scale (EPDS). The EPDS is a widely used, sensitive measure of both postnatal and prenatal depressive symptoms, with ten items each rated from 0 to 3 (higher scores indicate more depressive symptoms).^23,24^ Parental EPDS score during pregnancy was averaged based on the mean of three trimesters, whereas the EPDS score when the child was 12 months old was used as a single sum score. Continuous scores were used in the analyses.

#### Couple relationship satisfaction

Relationship satisfaction was assessed with the Revised Dyadic Adjustment Scale (RDAS), a 14-item version of the DAS24 (a short form of the Derriford Appearance Scale DAS59), measuring couple/partner adjustment in three domains.^25^ The items are rated on a scale from 1 to 6. Factor 1, termed ‘consensus’, consists of the items ‘career decisions’ and ‘religious matters’ (1, always agree; 2, almost always agree; 3, occasionally agree; 4, frequently disagree; 5, almost always disagree; 6, always disagree). Factor 2, termed ‘satisfaction’, consists of the items ‘How often do you discuss or have you considered divorce, separation or terminating your relationship?’ and ‘How often do you and your partner quarrel?’ (1, all of the time; 2, most of the time; 3, more often than not; 4, occasionally; 5, rarely; 6, never). Factor 3, termed ‘cohesion’, consists of the items ‘work together on a project’ and ‘calmly discuss something’ (1, never; 2, less than once a month; 3, once or twice a month; 4, once or twice a week; 5, once a day; 6, more often). The total scores range from 14 to 84, with higher scores representing lower levels of relationship satisfaction. Continuous sum scores of the RDAS scale at 34 gestational weeks and at 12 months postpartum were used in the present study.

#### Potential covariates and background factors

The following factors were included in the study as potential covariates. Paternal and maternal depressive symptoms and couple relationship satisfaction at 12 months, measured by EPDS and RDAS scales, were treated as potential covariates to rule out the possibility that the identified associations during pregnancy are explained by postnatal factors at the time of the RRI assessment. Educational level was based on parental report collected at gestational week 14, with responses categorised into low (secondary education or lower), medium (polytechnics/applied university degree) and high (university degree). Smoking status at offspring age of 3 months was measured based on dichotomic assessment (yes/no). Information about the number of siblings, length of gestation and child gender was obtained from the national birth register (www.thl.fi) after the delivery. The duration of breastfeeding (months) was measured as a cumulative exposure to breastfeeding, which was based on maternal reports at 3, 6 and 12 months postpartum, with the responses describing either full-time or part-time breastfeeding each month. Days on each part-time breastfeeding month were weighted as 0.5, and full-time month were weighted as 1, resulting in one cumulative score of ‘days of breastfeeding’.

Additionally, tympanostomy insertions and use of antibiotic treatments were included in the study as factors describing the study sample. Similar to RRIs, they were based on maternal reports by child age of 12 months.

### Statistical analyses

The analyses were performed with IBM SPSS 26.0 software for Windows. First, bivariate associations between paternal and maternal depressive symptoms, relationship satisfaction and child RRIs (presence of RRIs versus no RRIs, see the description above) were examined using independent sample *t*-tests. The associations between the potential covariates (length of gestation, child gender, number of siblings, breastfeeding until 12 months of age, parental education and child exposure to smoking) and RRIs were analysed using independent sample *t*-tests and *χ*²-tests. The missing data in the covariates (0%–4% in other covariates and 14.4% in the exposure to smoking variable), in the RDAS responses at 34 gestational weeks (1.7%–6.3%) and in the RDAS and EPDS responses at 12 months postpartum (3.5% in mothers and 26.5% in fathers) were multiple imputed using MCMC imputation procedure in SPSS, utilising ten imputation data-sets and 50 iterations. The pooled values across each analysis are reported; however, the analyses were also conducted with non-imputed data, with highly similar results as the imputed data.

#### Associations between the potential covariates and RRIs

Only the covariates statistically significantly (*P* < 0.05) associated with RRIs were included in the following models. The preliminary analyses with covariates (see Table 1) indicated that only paternal education (*χ*²[2 DF (degrees of freedom)] = 9.20, *P* = 0.011; lower education was associated with higher risk of RRI) and the number of siblings (*χ*²[5 DF] = 37.97, *P* < 0.001; infants with RRIs had more siblings than those with no RRIs) were associated with RRIs. Thus, these variables were included as covariates in the main models. Additionally, both paternal (*t* = −2.09, *P* = 0.021) and maternal (*t* = −2.66, *P* = 0.008) relationship satisfaction at 12 months postpartum were associated with child RRIs. By contrast, paternal depressive symptoms (*t* = −0.63, *P* = 0.534) and maternal depressive symptoms (*t* = −1.70, *P* = 0.089) at 12 months postpartum were not significantly associated with child RRIs. Postpartum symptoms were controlled for as described below.

**Table 1:** The sample characteristics (*N* = 766)

	Recurrent respiratory infections group (*n* = 50)	Comparison group (*n* = 716)	*P*-value^a^
	Mean (s.d.)	Mean (s.d.)	
Paternal age, years	33.2 (5.3)	32.7 (5.2)	0.520
Maternal age, years	31.9 (4.9)	30.9 (4.2)	0.104
Length of gestation, weeks	39.4 (1.48)	39.8 (1.6)	0.094
Cumulative exposure to breastfeeding, days	180.8 (78.0)	182.6 (78.5)	0.819
	*N* (%)		
Paternal education			0.011
Low	29 (58%)	269 (37%)	
Middle	7 (14%)	203 (28%)	
High	12 (24%)	213 (30%)	
Maternal education			0.473
Low	14 (28%)	173 (24%)	
Middle	14 (28%)	201 (28%)	
High	17 (34%)	320 (44%)	
Child gender (male)	28 (56%)	378 (53%)	0.660
Child exposure to parental smoking at 3 months	13 (26%)	144 (20%)	0.099
Number of siblings			<0.001
0	13 (26%)	416 (58%)	
1	21 (42%)	200 (28%)	
2	10 (20%)	80 (11%)	
≥3	6 (12%)	19 (3%)	
Tympanostomy tube insertions	11 (22%)	7 (1%)	<0.001
Antibiotic treatment			<0.001
0	7 (14%)	519 (73%)	
1–4	25 (50%)	192 (27%)	
5–10	18 (36%)	5 (1%)	
Infections by 12 months of age			<0.001
0	2 (4%)	403 (56%)	
1–4	22 (44%)	272 (38%)	
5–10	23 (46%)	40 (6%)	
>10	3 (3%)	1 (0.1%)	

*P*-values are calculated based on the imputed data-set, with highly similar findings in the non-imputed data-set.

a. Group comparisons.

#### Final models

A logistic regression analysis for paternal EPDS score and paternal RDAS score predicting child RRIs in four steps was conducted in the following manner: covariates listed above, with the exception of postpartum symptoms; model 1/2a, paternal EPDS/RDAS score during pregnancy; model 1/2b, maternal EPDS/RDAS score during pregnancy; and, if a pattern suggesting mediation was detected in model 1/2b, we then performed model 1/2c, including maternal EPDS/RDAS score at child age of 12 months (to rule out the possibility that all associations are explained by parental postnatal distress).

If no pattern suggesting mediation was found when conducting model 1/2b, then model 1/2c (controlling for postpartum effects) was not performed.

#### Mediation analysis

If there was an indication of a hypothesised mediation pattern in the logistic regression analysis (i.e. if the maternal symptoms during pregnancy were a significant predictor of child RRIs along with paternal symptoms), a mediation analysis was conducted with the Process MACRO v3.1. in SPSS.^26^ This analysis was run in the sample with no multiple imputation of covariates because of the software characteristics (*N* = 732, *N* = 48 and 6.6% with RRIs). In the mediation model, the indirect effect of the predictor (paternal symptoms) on RRIs through the mediator (maternal symptoms) was inspected. A two-sided *P* < 0.05 was regarded as significant.

## Results

### Sample characteristics and the association between the covariates and RRIs

Sample characteristics including sociodemographic and other background factors are presented in Table 1. Paternal education was associated with RRIs, with a greater number of children in the RRI group having a father with low education than the children in the comparison group. Maternal education was not related with RRIs within the sample. Further, greater number of siblings was associated with RRIs. Finally, up to 36% of children with RRIs had five or more antibiotic treatments before 12 months of age compared with 1% of children in the comparison group (*P* < 0.001). There was also a significant difference between the groups in the insertion of tympanic tubes (*P* < 0.001). Paternal or maternal age, length of gestation, cumulative exposure to breastfeeding, child gender and exposure to parental smoking were not significantly associated with RRIs. At 12 months postpartum, paternal depressive symptoms were not associated with RRIs, but lower relationship satisfaction was associated with a higher risk of RRIs. Maternal depressive symptoms and relationship satisfaction during pregnancy were also associated with child RRIs (see Table 2), a result that was reported in our earlier study with a larger sample of mother–child dyads.

**Table 2:** The associations between paternal and maternal symptoms during pregnancy and 12 months postpartum, and child recurrent respiratory infections

	Mean (pooled)		
	RRIs	Comparison group	*t-*value, *P*-value
	*n* = 50	*n* = 716	
Paternal			
EPDS score throughout pregnancy	4.33	3.32	−2.29, 0.022
RDAS score at 24 gestational weeks	33.80	30.32	−3.73, <0.001
EPDS score at 12 months postpartum	4.34	3.95	−0.63, 0.530
RDAS score at 12 months postpartum	35.40	32.57	−2.39, 0.021
Maternal			
EPDS score throughout pregnancy	6.53	4.48	−4.24, <0.001
RDAS score at 34 gestational weeks	32.56	29.79	−3.16, 0.002
EPDS score at 12 months postpartum	5.85	4.82	−1.70, 0.089
RDAS score at 12 months postpartum	34.71	32.08	−2.66, 0.008

RRIs, recurrent respiratory infections; EPDS, Edinburgh Postnatal Depression Scale; RDAS, Revised Dyadic Adjustment Scale.

### Bivariate associations between paternal and maternal psychological distress during pregnancy and child RRIs

This study builds on our earlier report on the associations between maternal prenatal distress and child RRIs.^8^ In this study with a smaller sample, the associations between both paternal and maternal distress and RRIs are displayed in Table 2. Paternal prenatal depressive symptoms and lower relationship satisfaction during pregnancy were associated with an elevated risk of RRIs (Table 2).

Furthermore, paternal and maternal depressive symptoms were modestly to moderately interrelated (EPDS score of *r* = 0.21 during pregnancy and *r* = 0.20 at 12 months postpartum; *P*s < 0.001). Relationship satisfaction among parents was moderately to highly correlated (*r* = 0.55 at 34 gestational weeks and *r* = 0.60 at 12 months postpartum; *P*s < 0.001).

### Logistic regression models for paternal depressive symptoms, relationship satisfaction and child RRIs at 12 months postpartum

The logistic regression models are displayed in Table 3. Paternal depressive symptoms were associated with an elevated risk of child RRIs (model 1a); however, this association diminished when the maternal depressive symptoms during pregnancy were controlled for, suggesting a mediation pattern through maternal symptoms (model 1b). The association between maternal depressive symptoms and child RRIs remained significant after controlling for 12-month postpartum maternal depressive symptoms (model 1c), and similar results were obtained when 12-month postpartum maternal relationship satisfaction was controlled for (Table 3).

**Table 3:** The logistic regression model for predicting child recurrent respiratory infections at 12 months of age

	*B*-value	*P*-value	Odds ratio	95% CI
Model 1a				
Paternal EPDS score	0.086	0.045	1.090	1.002–1.186
Model 1b				
Paternal EPDS score	0.059	0.191	1.061	0.971–1.159
Maternal EPDS score	0.116	0.004	1.123	1.039–1.214
Model 1c				
Paternal EPDS score	0.058	0.199	1.060	0.970–1.158
Maternal EPDS score	0.125	0.014	1.133	1.026–1.252
Postpartum control: maternal EPDS score at 12 months	−0.014	0.767	0.986	0.987–1.083
Model 2a				
Paternal RDAS score at 34 gestational weeks	0.062	0.004	1.06	1.020–1.110
Model 2b				
Paternal RDAS score at 34 gestational weeks	0.058	0.021	1.060	1.009–1.113
Maternal RDAS score at 34 gestational weeks	0.008	0.743	1.008	0.960–1.058
Model 2c				
Paternal RDAS score at 34 gestational weeks	0.053	0.041	1.054	1.002–1.109
Maternal RDAS score at 34 gestational weeks	–0.008	0.792	0.992	0.932–1.055
Postpartum control: paternal RDAS score at 12 months	0.026	0.396	1.027	0.966–1.091

Model a: paternal EPDS score, paternal education and number of siblings; model b: model 1 plus maternal EPDS score; model c: model 2 plus postpartum control (either maternal or paternal respective symptoms at 12 months postpartum). In addition, the models were also run across each predictor (paternal EPDS model controlled for maternal RDAS and paternal RDAS model controlled for maternal EPDS). EPDS, Edinburg Postnatal Depression Scale; RDAS, Revised Dyadic Adjustment Scale.

Lower paternal relationship satisfaction during pregnancy was similarly associated with higher risk of child RRIs (model 2a). However, this association remained even after controlling for maternal relationship satisfaction during pregnancy, which was not associated with child RRIs (model 2b), suggesting that poorer paternal relationship satisfaction is linked with child RRIs independently of maternal symptoms.

### Do maternal depressive symptoms and relationship satisfaction mediate the association between paternal distress and child RRIs?

**Fig. 2:**
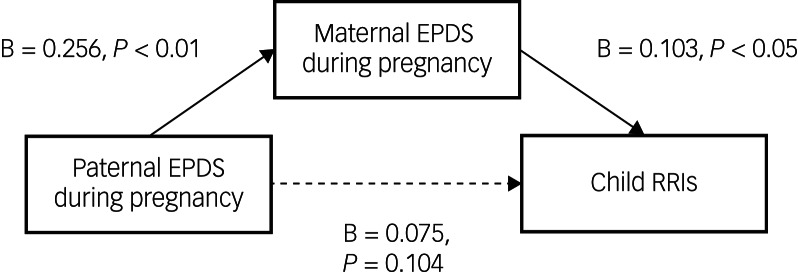
The mediation of paternal depressive symptoms and child RRIs through maternal depressive symptoms. EPDS, Edinburgh Postnatal Depression Scale; RRIs, recurrent respiratory infections.

The mediation model is reported in Fig. 2. After controlling for the same covariates as in the main models, maternal depressive symptoms during pregnancy indirectly mediated the association between paternal depressive symptoms during pregnancy and child RRIs (*B* = 0.0263, s.e. = 0.013, 95% bootstrapped CI 0.003–0.055).

## Discussion

Our study showed that paternal depressive symptoms and lower couple relationship satisfaction in pregnancy are associated with a higher risk of child RRIs. Our primary hypothesis was partially supported as maternal prenatal depressive symptoms indirectly mediated the association between paternal prenatal depressive symptoms and child RRIs. Nevertheless, we also found a direct association between lower paternal couple relationship satisfaction and a higher risk of child RRIs, and this seems to be independent of maternal prenatal couple relationship satisfaction. To our knowledge, this is one of the first studies investigating the role of paternal psychological distress in offspring RRI risk, and its mediation through maternal well-being during pregnancy. Our study indicates differential pathways through which paternal psychological distress may be linked with offspring health outcomes in the first years of life.

Our findings regarding the association between paternal prenatal psychological distress and offspring RRIs suggest a link with foetal programming and its potential effects on the development of the child's immune system. The hypothalamic-pituitary-adrenal (HPA) axis and the immune system work in close interaction.^27^ Alterations in the HPA axis induced by maternal distress and the related biological signals in the intrauterine environment are well documented, although the exact mechanisms are yet to be confirmed.^28,29^ An increasing body of research has reported on the link between maternal distress during pregnancy and offspring immunity and proneness to infections.^9,10^ However, along with few prior reports, the current study highlights the role of fathers as an additional contributor to this cascade, as paternal depressive symptoms are related to child infections through maternal well-being.^30,31^ Thus, the results strengthen the need to screen paternal/partner mental health during pregnancy along with maternal well-being, and highlight the possibility of preventing child adverse health outcomes by supporting not only maternal, but also paternal and whole family, psychosocial well-being during pregnancy.

In turn, we detected an unexpected direct association between paternal couple relationship satisfaction and child RRIs at 12 months of age. This association was independent of maternal relationship satisfaction, which we considered to be the primary transmission route for prenatal psychosocial stressors, including the well-being of the father. It is possible that there are other mother-related transmission routes that were not examined in the current study, such as maternal HPA axis functioning, other aspects of maternal psychosocial distress (such as social support) and lifestyle factors (such as diet).^32,33^ However, recent research has also suggested that fathers may have an independent role in transmitting the risk for disease, and that this route may be preconceptional, transmitted through paternal germline effects.^34^ Furthermore, fathers who experience lower relationship satisfaction are more likely to have experienced other risk factors, such as childhood maltreatment, and these exposures may be related to epigenetic changes in paternal germline and, consequently, could lead to biological alterations in the offspring, including functioning of stress regulation and the immune system.^32,35,36^ Key mechanisms for how a father's environmental exposure is passed on to the next generation are non-genetic alterations within sperm, such as small non-coding ribonucleic acids, DNA damage, DNA methylation and histone modifications.^34^

For example, childhood maltreatment may precede conflicts and poorer relationship satisfaction in adulthood relationships both in women and men,^37,38^ and thus explain why relationship satisfaction in the current study was independently associated with child RRIs even when maternal well-being and couple satisfaction was accounted for. However, these explanations remain preliminary and require further investigation into whether such mechanisms can be detected in terms of child RRIs and other physical health outcomes.

One relevant factor when putting these findings into context are other psychosocial distress-related processes that can be contributors for child RRIs – factors we attempted to control for in our analyses. Both in our study and in previous literature, parental lower relationship satisfaction during pregnancy and postpartum were found to be associated with a higher risk of child RRIs.^39^ As discussed above, the parental relationship is a source of psychological support for both adults. Distress and dissatisfaction within the family may also increase the frequency of asking for medical support even in the case of usual respiratory infections that would not otherwise need medical intervention. This, in turn, may lead to unnecessary antibiotic treatments, which have consequences for child microbiota and future proneness to infections. This explanation emphasises provision of psychosocial support for the family and the couple's relationship from pregnancy onward, as one effective way to prevent child respiratory infections, especially if targeted to the prenatal and early postnatal period. In this study, we focused primarily on the mediating effects of maternal psychological distress between paternal well-being and child RRIs. However, although beyond the scope of the present study, interesting research questions also include the possible effects of family dynamics on RRI risk, for instance, whether paternal stress moderates the association between maternal distress and child RRIs, and whether there are important timing effects concerning such dynamics. Furthermore, more intense research designs (for instance, economic momentary assessments of parental distress) would be needed to determine timing effects concerning the role of the prenatal and postnatal period and which trimester of pregnancy proves most powerful in driving the detected associations.

The main strengths of this study include a prospective study design that allowed for longitudinal measurements during pre- and postnatal periods, with extensive measurements of psychosocial stress with validated questionnaires. Second, both maternal and paternal self-reports were used to study associations with offspring RRIs in parallel. The main limitation is the use of self-reports in the assessment of RRIs. Parents with higher psychological distress may also report a higher number of infections than parents with lower distress because child physical illness is perceived as more demanding. However, our RRI group clearly differed from the control group regarding the number of infections and antibiotic treatments, which strengthens the reliability of our outcome measure and thus the findings of our study in general. Another limitation is that within the scope of this study, we were not able to examine the timing effect of prenatal maternal stress. The timing of prenatal maternal stress may affect foetal programming for the development of both the lungs and the immune system.

In conclusion, in the context of foetal programming and its effects on child health, paternal exposures should be acknowledged and adequately screened in paediatric clinical settings. The results also indicate different pathways through which fathers’ distress during pregnancy may contribute to elevated risk of offspring RRIs. Paternal well-being during pregnancy may be transmitted to offspring through maternal well-being, but there may also be novel mechanisms independent of maternal transmission. Future research should investigate these mechanisms and biological mediators in more detail.

## Data Availability

The data that support the findings of this study are available from the corresponding author, L.K., upon reasonable request.

## References

[1] Fendrick AM, Monto AS, Nightengale B, Sarnes M. The economic burden of non–influenza-related viral respiratory tract infection in the United States. Arch Intern Med 2003; 163(4): 487. 12588210 10.1001/archinte.163.4.487

[2] Toivonen L, Karppinen S, Schuez-Havupalo L, Teros-Jaakkola T, Vuononvirta J, Mertsola J, et al. Burden of recurrent respiratory tract infections in children. Pediatr Infect Dis J 2016; 35(12): e362–9. 27455443 10.1097/INF.0000000000001304

[3] Jiang X, Sun L, Wang B, Yang X, Shang L, Zhang Y. Health-related quality of life among children with recurrent respiratory tract infections in Xi'an, China. PLoS One 2013; 8(2): e56945. 23451114 10.1371/journal.pone.0056945 PMC3581567

[4] Jedrychowski W, Flak E. Maternal smoking during pregnancy and postnatal exposure to environmental tobacco smoke as predisposition factors to acute respiratory infections. Environ Health Perspect 1997; 105(3): 302–6. 9171991 10.1289/ehp.97105302 PMC1470010

[5] Nicolai A, Frassanito A, Nenna R, Cangiano G, Petrarca L, Papoff P, et al. Risk factors for virus-induced acute respiratory tract infections in children younger than 3 years and recurrent wheezing at 36 months follow-up after discharge. Pediatr Infect Dis J 2017; 36(2): 179–83. 27798551 10.1097/INF.0000000000001385

[6] Sonego M, Pellegrin MC, Becker G, Lazzerini M. Risk factors for mortality from acute lower respiratory infections (ALRI) in children under five years of age in low and middle-income countries: a systematic review and meta-analysis of observational studies. PLoS One 2015; 10(1): e0116380. 25635911 10.1371/journal.pone.0116380 PMC4312071

[7] Tegethoff M, Greene N, Olsen J, Schaffner E, Meinlschmidt G. Stress during pregnancy and offspring pediatric disease: a national cohort study. Env Heal Perspect 2011; 119(11): 1647–52. 10.1289/ehp.1003253 PMC3226491 21775267

[8] Korhonen LS, Karlsson L, Scheinin NM, Korja R, Tolvanen M, Mertsola J, et al. Prenatal maternal psychological distress and offspring risk for recurrent respiratory infections. J Pediatr 2019; 208: 229–35.e1. 10.1016/j.jpeds.2018.12.050 30723014

[9] Rubin LP. Maternal and pediatric health and disease: integrating biopsychosocial models and epigenetics. Pediatr Res 2016; 79(1–2): 127–35. 26484619 10.1038/pr.2015.203

[10] Pierce M, Hope HF, Kolade A, Gellatly J, Osam CS, Perchard R, et al. Effects of parental mental illness on children's physical health: systematic review and meta-analysis. Br J Psychiatry 2020; 217(1): 354–63. 31610824 10.1192/bjp.2019.216

[11] Paulson JF, Bazemore SD. Prenatal and postpartum depression in fathers and its association with maternal depression: a meta-analysis. JAMA 2010; 303(19): 1961–9. 20483973 10.1001/jama.2010.605

[12] Schor EL, American Academy of Pediatrics Task Force on the Family. Family pediatrics: report of the Task Force on the Family. Pediatrics 2003; 111(6 Pt 2): 1541–71. 12777595

[13] Davies PT, Forman EM. Children's patterns of preserving emotional security in the interparental subsystem. Child Dev 2002; 73(6): 1880–903. 12487500 10.1111/1467-8624.t01-1-00512

[14] Erel O, Burman B. Interrelatedness of marital relations and parent-child relations: a meta-analytic review. Psychol Bull 1995; 118(1): 108–32. 7644602 10.1037/0033-2909.118.1.108

[15] Schuez-Havupalo L, Lahti E, Junttila N, Toivonen L, Aromaa M, Rautava P, et al. Parents’ depression and loneliness during pregnancy and respiratory infections in the offspring: a prospective birth cohort study. PLoS One 2018; 13(9): e0203650. 30192872 10.1371/journal.pone.0203650 PMC6128609

[16] Guxens M, Sonnenschein-Van Der Voort AMM, Tiemeier H, Hofman A, Sunyer J, De Jongste JC, et al. Parental psychological distress during pregnancy and wheezing in preschool children: the generation R study. J Allergy Clin Immunol 2014; 133(1): 59–67.e12. 23777854 10.1016/j.jaci.2013.04.044

[17] Magnus MC, Wright RJ, Røysamb E, Parr CL, Karlstad Ø, Page CM, et al. Association of maternal psychosocial stress with increased risk of asthma development in offspring. Am J Epidemiol 2018; 187(6): 1199–209. 29244063 10.1093/aje/kwx366 PMC5982733

[18] Van Meel ER, Saharan G, Jaddoe VWV, De Jongste JC, Reiss IKM, Tiemeier H, et al. Parental psychological distress during pregnancy and the risk of childhood lower lung function and asthma: a population-based prospective cohort study. Thorax 2020; 75(12): 1074–81. 33046570 10.1136/thoraxjnl-2019-214099 PMC7677473

[19] Sweeney S, MacBeth A. The effects of paternal depression on child and adolescent outcomes: a systematic review. J Affect Disord 2016; 205: 44–59. 27414953 10.1016/j.jad.2016.05.073

[20] Hughes C, Devine RT, Mesman J, Blair C. Parental well-being, couple relationship quality, and children's behavioral problems in the first 2 years of life. Dev Psychopathol 2020; 32(3): 935–44. 31339479 10.1017/S0954579419000804

[21] Kim P. How stress can influence brain adaptations to motherhood. Front Neuroendocrinol 2021; 60: 100875. 33038383 10.1016/j.yfrne.2020.100875 PMC7539902

[22] Karlsson L, Tolvanen M, Scheinin NM, Uusitupa H-M, Korja R, Ekholm E, et al. Cohort profile: the FinnBrain birth cohort study (FinnBrain). Int J Epidemiol 2018; 47(1): 15–16j. 29025073 10.1093/ije/dyx173

[23] Cox JL, Holden JM, Sagovsky R. Detection of postnatal depression. development of the 10-item Edinburgh Postnatal Depression Scale. Br J Psychiatry 1987; 150(6): 782–6. 10.1192/bjp.150.6.782 3651732

[24] Bergink V, Kooistra L, Lambregtse-van den Berg MP, Wijnen H, Bunevicius R, van Baar A, et al. Validation of the Edinburgh Depression Scale during pregnancy. J Psychosom Res 2011; 70(4): 385–9. 21414460 10.1016/j.jpsychores.2010.07.008

[25] Busby DM, Christensen C, Crane DR, Larson JH. A revision of the Dyadic Adjustment Scale for use with distressed and nondistressed couples: construct hierarchy and multidimensional scales. J Marital Fam Ther 1995; 21(3): 289–308.

[26] Hayes AF. Introduction to Mediation, Moderation, and Conditional Process Analysis: A Regression-Based Approach in Series Methodology in the Social Sciences (2nd edn). Guilford Press, 2017.

[27] Elwenspoek MMC, Kuehn A, Muller CP, Turner JD. The effects of early life adversity on the immune system. Psychoneuroendocrinology 2017; 82: 140–54. 28549270 10.1016/j.psyneuen.2017.05.012

[28] Van den Bergh BRH, van den Heuvel MI, Lahti M, Braeken M, de Rooij SR, Entringer S, et al. Prenatal developmental origins of behavior and mental health: the influence of maternal stress in pregnancy. Neurosci Biobehav Rev 2020; 117: 26–64. 28757456 10.1016/j.neubiorev.2017.07.003

[29] Creutzberg KC, Sanson A, Viola TW, Marchisella F, Begni V, Grassi-Oliveira R, et al. Long-lasting effects of prenatal stress on HPA axis and inflammation: a systematic review and multilevel meta-analysis in rodent studies. Neurosci Biobehav Rev 2021; 127: 270–83. 33951412 10.1016/j.neubiorev.2021.04.032

[30] Xiong F, Zhang L. Role of the hypothalamic-pituitary-adrenal axis in developmental programming of health and disease. Front Neuroendocrinol 2013; 34: 27–46. 23200813 10.1016/j.yfrne.2012.11.002 PMC3594480

[31] Brew BK, Lundholm C, Viktorin A, Lichtenstein P, Larsson H, Almqvist C. Longitudinal depression or anxiety in mothers and offspring asthma: a Swedish population-based study. Int J Epidemiol 2018; 47(1): 166–74. 29040553 10.1093/ije/dyx208 PMC5837783

[32] O'Donnell KJ, Meaney MJ. Fetal origins of mental health: the developmental origins of health and disease hypothesis. Am J Psychiatry 2017; 174(4): 319–28. 27838934 10.1176/appi.ajp.2016.16020138

[33] Beijers R, Buitelaar JK, de Weerth C. Mechanisms underlying the effects of prenatal psychosocial stress on child outcomes: beyond the HPA axis. Eur Child Adolesc Psychiatry 2014; 23(10): 943–56. 24875898 10.1007/s00787-014-0566-3

[34] Linschooten JO, Verhofstad N, Gutzkow K, Olsen AK, Yauk C, Oligschläger Y, et al. Paternal lifestyle as a potential source of germline mutations transmitted to offspring. FASEB J 2013; 27: 2873–9. 23538710 10.1096/fj.13-227694 PMC3688758

[35] DiLillo D, Peugh J, Walsh K, Panuzio J, Trask E, Evans S. Child maltreatment history among newlywed couples: a longitudinal study of marital outcomes and mediating pathways. J Consult Clin Psychol 2009; 77(4): 680–92. 19634961 10.1037/a0015708

[36] Rodgers AB, Morgan CP, Bronson SL, Revello S, Bale TL. Paternal stress exposure alters sperm microRNA content and reprograms offspring HPA stress axis regulation. J Neurosci 2013; 33(21): 9003–12. 23699511 10.1523/JNEUROSCI.0914-13.2013 PMC3712504

[37] Zamir O. Childhood maltreatment and relationship quality: a review of type of abuse and mediating and protective factors. Trauma Violence Abuse 2022; 23(4): 1344–57. 33657928 10.1177/1524838021998319

[38] Liu S, Wang Z, Lu S, Shi J. Dyadic analysis of childhood emotional maltreatment and marital satisfaction during the transition to parenthood: the mediating effects of emotion regulation strategies and psychological distress. J Aggress Maltreat Trauma 2019; 28(10): 1216–31.

[39] Henriksen RE, Thuen F. Marital quality and stress in pregnancy predict the risk of infectious disease in the offspring: the Norwegian mother and child cohort study. PLoS One 2015; 10(9): e0137304. 10.1371/journal.pone.0137304 PMC4589358 26422017

